# Large-scale fMRI dataset for the design of motor-based Brain-Computer Interfaces

**DOI:** 10.1038/s41597-025-05134-1

**Published:** 2025-05-16

**Authors:** Magnus S. Bom, Annette M. A. Brak, Mathijs Raemaekers, Nick F. Ramsey, Mariska J. Vansteensel, Mariana P. Branco

**Affiliations:** Department of Neurology and Neurosurgery, UMC Utrecht Brain Center, University of Utrecht, Utrecht, the Netherlands

**Keywords:** Neurology, Paediatric research

## Abstract

Functional Magnetic Resonance Imaging (fMRI) data is commonly used to map sensorimotor cortical organization and to localise electrode target sites for implanted Brain-Computer Interfaces (BCIs). Functional data recorded during motor and somatosensory tasks from both adults and children specifically designed to map and localise BCI target areas throughout the lifespan is rare. Here, we describe a large-scale dataset collected from 155 human participants while they performed motor and somatosensory tasks involving the fingers, hands, arms, feet, legs, and mouth region. The dataset includes data from both adults and children (age range: 6–89 years) performing a set of standardized tasks. This dataset is particularly relevant to study developmental patterns in motor representation on the cortical surface and for the design of paediatric motor-based implanted BCIs.

## Background & Summary

Brain Computer Interfaces (BCIs) are devices that can convert brain signals to commands to control a computer, providing for example severely paralysed individuals with capabilities they have lost due to their paralysis. Specifically, communication BCIs aim to address severe loss of communication^1^. For implanted communication BCIs^2–6^ the precise identification of electrode target areas prior to implantation is crucial for optimal performance. Functional magnetic resonance imaging (fMRI) has been proposed as a suitable tool for pre-implantation cortical mapping and electrode target localization for implanted BCIs^7^, due to its non-invasive properties, high spatial resolution, and high correlation with BCI performance^8–12^.

For BCIs that are controlled by (attempted) movements, signals are usually extracted from the primary sensorimotor cortex (SMC). Common SMC regions for extraction of BCI control signals are the hand, mouth, and foot areas. Many studies have investigated activation patterns in healthy young adults during simple tasks involving hand or finger movement^13–17^. Such tasks have been found to robustly activate the hand and finger area of the SMC^18^. These findings extend into aging populations^19,20^, and provide a good basis for motor based BCI use in adults.

Motor-based BCIs could also potentially be used to help children with severe communication impairment due to for example Cerebral Palsy. Considerations for implanting BCIs in children is gaining attention in the field of BCIs^21–24^. Yet, implementing BCIs in children poses challenges separate from those in adults. For example, little research has been dedicated to identifying developmental changes in primary motor cortex (M1) activation from childhood into adulthood. Some studies showed consistent activation of the contralateral SMC in children^25–27^ but there may be differences in activation patterns between age groups that could have implications for the design and longitudinal use of implanted BCIs across the lifespan. For example, implanting electrodes at a site that can drive a BCI during childhood but cannot sustain the use of the BCI into adulthood would require re-insertion of the implanted electrodes at an older age, leading to additional surgical risk.

While large fMRI datasets are available to the research community^28–30^, there are to our knowledge few available datasets that use motor tasks that can be used to map and localise sensorimotor areas for BCI control, and of which the motor output is not used merely as a metric of attention (e.g., button pressing). fMRI datasets are also available for both children^31^ and adults^29,30,32^, but they commonly include data from either children or adults, often with different tasks, and thus generally do not allow for comparison between age groups.

Here we present the first large-scale fMRI dataset of 155 children and adults performing a standardized set of motor and somatosensory tasks specifically designed for pre-implantation localization of sensorimotor function. The dataset includes a total of 471 runs involving the hand and fingers, tongue, as well as other limbs, such as the arms or legs. This dataset is valuable to the cross-sectional study of key topics related to the feasibility of BCIs in children and adults, such as assessing the presence of a somatotopic map of the hand and other body parts, or the influence of brain development and aging on the somatotopic map. The addition of a somatosensory stimulation task allows to further study the potential cortical overlap between execution/imagined movement and proprioceptive/tactile feedback. Moreover, age-group-level results may also be capitalized to predict for example the best electrode configuration for BCI implants^12^. Outside of the BCI domain, this dataset is valuable for the investigation of parameters such as the structural development and aging of the brain, and general body mapping across age groups. By making this dataset publicly available, we hope to promote research on the feasibility of implanted BCIs in children and young adults, allowing researchers to address gaps in the literature related to the representation of brain function in the sensorimotor areas from childhood into adulthood relevant to BCI control.

## Methods

### Participants

**Fig. 1:**
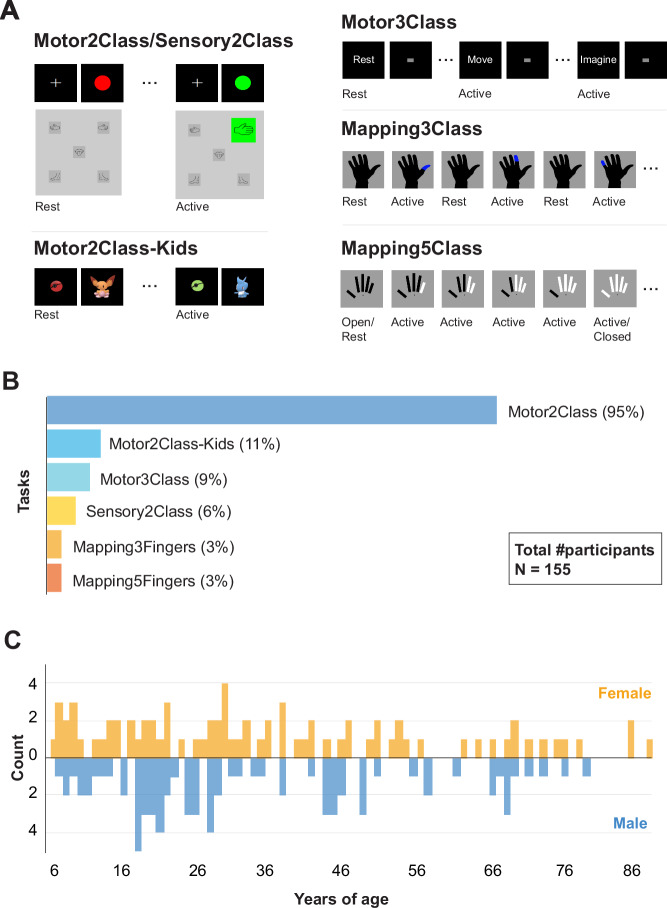
Overview of tasks and demographics. (**A**) Illustration of task paradigms for Motor/Sensory2Class, Motor2ClassKids, Motor3Class, Mapping3Class and Mapping5Class. A short video of each task can be found in the folder ‘task_examples’. (**B**) Percentage of participants who performed each task out of total of 155 participants. (**C**) Histogram of age distribution for females (yellow) and males (blue) in the dataset.

The dataset was obtained from several studies performed at the University Medical Centre Utrecht over the last 15 years. Data was collected from 155 participants (mean age: 35.5±21.3, range: 6–89, 49.7% (78) females, 88.5% (139) right-handed and 1.9% (3) ambidextrous, Fig. 1c, Supplementary Table S1) who performed 471 tasks in total. Some participants (N = 63) were admitted to the hospital for diagnostic procedures related to their medication-resistant epilepsy (N = 60) or surgical removal of a tumour (N = 3, sub-16, −21 and −38). As part of the pre-surgical workup, these patients underwent fMRI recordings and participated in fMRI sensorimotor experiments for clinical or research purposes. Other participants were healthy volunteers (N = 92) and participated in studies targeting functional mapping of movement. All participants gave written informed consent to participate in the research for which data was acquired and for the use of their data for research purposes. For participants under the age of 18, the informed consent was obtained from the participant’s parents and/or legal guardian. If they were older than 12, these participants also signed an informed consent form. Given that the data is fully anonymous (defaced, randomized and without key) and shared using the BIDS format (https://bids.neuroimaging.io/)^33^, no extra consent was required according to applicable rules and regulations in the Netherlands.

### Experimental procedures

Data was collected during performance of one of six different standardized tasks specifically designed for sensorimotor localization of hands, mouth or feet (Fig. 1a): ‘Motor2Class’ (148 participants), ‘Motor2ClassKids’ (17 participants), ‘Sensory2Class’ (10 participants), ‘Motor3Class’ (14 participants), ‘Mapping3Fingers’ (4 participants), and ‘Mapping5Fingers’ (5 participants) (Fig. 1b). A total of 471 runs were acquired, of which 88.32% (416) consisted of ‘Motor2Class’. All participants completed at least one task, and some participants completed multiple tasks and/or multiple runs of the same task with multiple body parts.

### Motor2Class and sensory2class

The Motor2Class and Sensory2Class were block design tasks with two conditions: rest and active (Fig. 1a). During the ‘rest’ condition the participant was asked to lie as still as possible while fixating at the center of a screen. During the ‘active’ condition, the participant was asked to either continuously move a body part (Motor2Class) or to rest while a body part would be stimulated with a brush by a researcher (Sensory2Class). For all Sensory2Class and most Motor2Class tasks, the stimulus presentation included a black screen with blinking circles cuing active (green circle) or rest (red circle) blocks. For the majority of participants, each block lasted for 30 s, with a full task consisting of 4 active blocks and 5 rest blocks for 3T and 7T scans, and 8 active blocks and 9 rest blocks for 1.5 T scans. For a few Motor2Class scans (sub-9, 66 and 155), each block had varying durations (13–43 s) with a full task consisting of 7 active blocks and 8 rest blocks. One participant performed 8 active blocks and 9 rest blocks, with active blocks lasting for 30 s. For other participants (sub-79-154, all 3T) an alternative block-design task was used for motor localization, where pictures of body parts were depicted on a screen, in grey during rest blocks. In this design, a full task consisted of 5 active blocks and 6 rest blocks or 7 active blocks and 8 rest blocks of varying durations (8–43 s and 7–44 s). During active blocks the body part that was requested to move was blinking in green. The body parts included in this dataset using the above tasks were thumb, index or little fingers, all fingers in a ‘fingertapping’ fashion (‘fingers’), hand (open/close movement), arm, feet (both feet simultaneously), lip (kissing movement), tongue (left-right movement) and other general mouth movements (Supplementary Table 1). The tasks were carried out with either the right or the left body part, except in the case of feet, lip, tongue and mouth movements. Somatosensory stimulation was performed with a (large round) brush on the whole palm of the hand or sole/top of the foot, with continuous movements during the active block from wrist to tip of fingers or ankle to tip of toes (and back), respectively.

### Motor2ClassKids

The Motor2ClassKids is child-friendly version of the Motor2Class task (Fig. 1a). This task was designed to engage children in the task and thus improve the quality of the data^34^. Similarly to Motor2Class, the block design task consisted of alternating ‘rest’ and ‘active’ blocks. During the ‘rest’ condition the child was asked to lay as still as possible and look at pictures of a cartoon alternating with a picture of a red ‘ball’. During the ‘active’ block, a green ‘ball’ blinked alternating with a ‘cartoon character’ cuing the movement. The participant was instructed to squeeze of a rubber-bulb with the hand every time a ‘cartoon character’ would appear on the screen (body part ‘hand’ in Supplementary Table 1). Each squeeze provided feedback to the participant by displaying a coloured line around the image (i.e., the ‘cartoon character’ was caught with the ‘ball’). A complete run consisted of 5 active blocks and 5 rest blocks (22–30 s duration) each containing 11 ‘cartoon characters’. This task was performed by participants aged between 6 and 11 years old, who also performed a Motor2Class task. For more details regarding the task, protocol and inclusion criteria, and associated results see^34^. Results of both Motor2Class and Motor2ClassKids can be compared and/or combined in order to estimate adequate cortical mapping for paediatric BCI applications.

### Motor3Class

The Motor3Class task is an alternative block design task with three conditions: one rest and two active conditions, namely overtly/executed movements and imagined movements (Fig. 1a). Each condition was cued with an instruction ‘rest’, ‘move’ or ‘imagine’, followed by a block with a blinking grey square. In the ‘rest’ condition the participant was asked to lay as still as possible while fixating on the screen. During the active conditions the participant was asked to continuously move the pre-specified body part (see Table 1). During the ‘move’ blocks, the participant was required to execute movements overtly, while during the ‘imagine’ blocks the participant imagined performing the same movements. There were a total of 10 blocks per condition, and each block had a duration of 17 s including a 1.3 s instruction screen (for more details see^35^. The body parts included in the dataset were either movement of all fingers in a ‘fingertapping’ fashion (‘fingers’), foot or tongue (Supplementary Table 1). The movement or imagination of movement involved the right or the left body part, except for the tongue.

### Mapping3Fingers

The Mapping3Fingers task mapped three individual fingers: thumb, index and little fingers using an event-related design (Fig. 1a). Each movement consisted of two flexions of the specified finger. A black contour of a hand was displayed on a screen. The movement of the fingers was cued by highlighting the thumb, little finger or index finger in blue for 0.5 s. The task consisted of 30 cues of each finger presented in randomized order with an inter-trial interval of 4.4 s (for more details see^36^).

### Mapping5Fingers

The Mapping5Fingers task also used an event-related design to map five individual fingers (Fig. 1a): thumb, index finger, middle, ring and little finger. In this task flexion and extension of fingers was independently cued, and fingers were cued individually and sequentially. Five rectangles representing each of the five fingers were displayed on a screen. The rectangle turned white to cue finger flexion, and black to cue finger extension. Each finger was kept in the same position (flexed or extended) until the movement of that same finger was cued again. Each finger was cued 8 times during the run at 4.8 s intervals, except for the first and last finger which had a longer interval of 14.4 s (rest block). Participants started the experiment with an open palm (for more details see^15^).

### Data acquisition details

#### Structural data acquisition

Structural images of the whole brain were acquired on either a 1.5 T ACS-NT Philips scanner (N = 8 participants), a 3 T Achieva Philips scanner (N = 139 participants) or a 7 T Achieva Philips scanner (N = 13 participants). Some participants had multiple structural scans, one per session. The scanning resolution varied between 0.5 and 2 mm. The specific parameters are available for each scan in the JSON sidecar files and the NIfTI (*.nii) file headers of the dataset.

#### fMRI data acquisition

Functional images were acquired on either a 1.5 T ACS-NT Philips scanner (N = 20 scans), a 3 T Achieva Philips scanner (N = 434 scans) or a 7 T Achieva Philips scanner (N = 17 scans). A PRESTO scanning sequence was used for the 1.5 T scans and 3 T scans, which allows for fast full brain coverage by means of echo shifting^37^. An EPI scanning sequence was used for the 7 T scans. Whole brain scans were acquired for all scans except for some 1.5 T and all 7 T scans (Supplementary Figure S1), where a limited field of view was used that included the dorsal part of the brain, extending ventrally to include the hand and finger area. Lower parts of brainstem and cerebellum were often not included. The acquisition time per volume ranged between 0.5 and 4.86 s, and the voxel size between 1.384 × 1.384 × 1.5 and 4.5 × 4 × 4 mm. The specific parameters including the scanning sequence are available for each scan in the JSON (*.json) metadata sidecar files and the NIfTI (*.nii) file headers of the dataset. Dummy scans were excluded by the scanner. Synchronization varied across tasks with triggering per trial for event-related designs and triggering per block or single trigger at the start of the task for blocked designs. Synchronization events always excluded dummy scans.

## Data Records

The dataset is available at the open public repository OpenNeuro (ds005366)^38^ and it can be downloaded from the link 10.18112/openneuro.ds005366.v2.0.0. Data is shared under the C00 public domain license.

### De-identification and defacing of structural images

All personal identifiable information has been removed from the data. All individual structural images were defaced to comply with the requirements for sharing de-identified medical data. The images were defaced using SPM12 (https://www.fil.ion.ucl.ac.uk/spm/)^39^.

### Conversion to BIDS structure

**Fig. 2:**
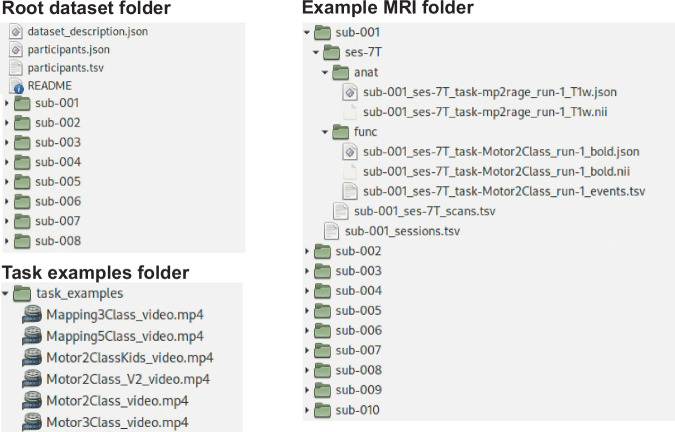
Overview of BIDS dataset. Structure of files and sub-folders in the root dataset folder and inside subject 001 (sub-001) folder. Each subject folder contains one or more (MRI) sessions with a folder for structural (‘anat’) and functional (‘func’) data. Overview of the task example folder, where short videos of the tasks are saved.

Data was standardized using Python (version 3.9) and MATLAB (The MathWorks Inc., 2022) tools. Raw PAR/REC (f)MRI files were converted to NIfTI format using the Nibabel library^40^. PAR/REC is a format generated by Philips scanners. Data was converted to Brain Imaging Data Structure (BIDS) using the function data2bids.m from Fieldtrip Toolbox^41^. The dataset is therefore organized according to the BIDS format (Fig. 2): the root folder contains metadata about the description of the dataset (dataset_description.json), the list of participants along with their demographic details (participants.tsv), a folder with task examples (task_examples), and individual data folders per participant named sub-XXX. The order in which participants were saved in the dataset was randomised (sub-79 to 154 were already randomized before being added to this dataset) and the randomisation key has been deleted, making this dataset fully anonymous. The dataset was validated using https://bids-standard.github.io/bids-validator/.

### Participant data folders

Each participant’s folder contains one or two sub-directories depending on how many scanning sessions they participated in (Fig. 2), typically corresponding to the field strength of the MRI scanner used during the session (ses-1.5T, ses-3T, or ses-7T). Some participants performed two sessions at 1.5T, and so the folders are referred to ses-1.5T and ses-1.5T2, for session 1 and 2 respectively.

### (f)MRI folders

Inside each session folder, there are two sub-directories (Fig. 2): a folder containing one or multiple anatomical MRI scans (‘anat’), and a folder containing one or multiple functional MRI scans (‘func’). Each participant has an anatomical MRI folder in at least one of their session folders. (f)MRI data are provided in the NIfTI (*.nii) format with sidecar JSON (*.json) files that store additional metadata. Functional images are accompanied by a TSV (*events.tsv) file that contains onsets, event durations (in seconds) and the type of the events of the motor tasks.

### Task examples folder

We additionally added a folder containing short videos of each of the five task paradigms (Fig. 2). The videos show one of each block type for block designed tasks (Motor2Class, Motor2ClassKids, Sensory2Class and Motor3Class), and roughly 1 minute of the task for event-related designs (Mapping3Fingers and Mapping5Fingers). Motor2Class and Sensory2Class share the same video, as they have identical visual stimuli (see Fig. 1a).

## Technical Validation

Data that had only partial coverage of the sensorimotor cortex, had no event-logs or had identifiable brain anomalies were excluded before BIDS conversion. The remaining data was processed with SPM12 and Freesurfer^42^. Preprocessing steps of functional images included realignment, unwarping, and coregistration of the functional images to the anatomical images. Note that no denoising was performed. Surface reconstructions of the anatomical images were created using the Freesurfer recon-all pipeline and the functional images were mapped to the ‘fsaverage’ surface while using a smoothing kernel of 6 mm FWHM. Basic data quality was assessed using a two-step approach (see details below): 1) we analysed head motion; and 2) we correlated activation patterns of individual scans with the mean activation pattern of other participants that performed the same task using the same limb and performed visual inspections of the activation patterns; subsequently, we performed visual inspection of the poorly correlated activation patterns. Based on these two metrics we rated each run. Data included in the dataset was deemed good after these two steps as detailed below. For completeness, we also include the temporal signal-to-noise ratio per scanner type (see Supplementary Figure S2). Last, for illustration purposes we computed the activation pattern in response to the Motor2Class ‘fingers’ task across all participants that performed this task. Of note, we performed a basic data quality analysis on the dataset, but more advanced scan quality assessment, such as calculating ratios between BOLD activity between sensorimotor cortex and other cortical regions, may also be performed.

### Analysis of head motion

**Fig. 3:**
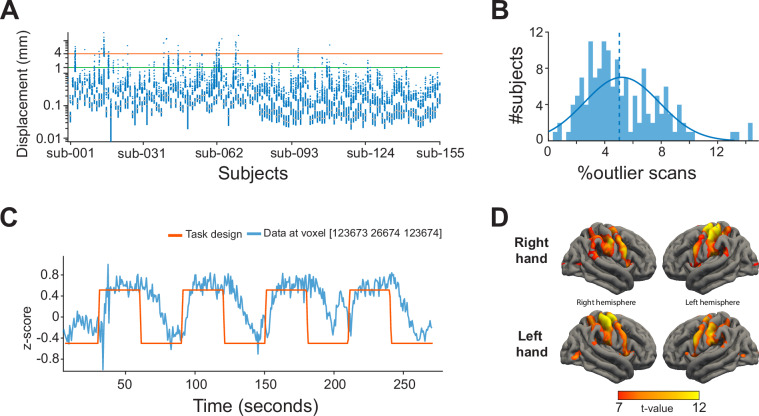
Technical data validation. (**A**) Boxplot of the framewise displacement metric per subject averaged over scans with maximum and minimum voxel sizes for the dataset marked by green and red horizontal lines, respectively. (**B**) Histogram number of participants with percentage of volumes classified as motion outliers. (**C**) Example time course of the Motor2Class task and observed fMRI activity in one voxel of one subject. (**D**) Second-level group statistics of hand movements from the Motor2Class using ‘fingers’ (fingertapping) for all participants combined, using the t-values on the 90th percentile.

Analysis of head motion involved assessment of the framewise displacement based on the transformation matrices produced by SPM during realignment and unwarping of the raw NIfTI files. The x, y, and z displacement for each voxel and each volume was calculated based on the changes in the transformation matrices following realignment. In addition, motion outliers were calculated independently using FSL (https://fsl.fmrib.ox.ac.uk/; default settings)^43,44^. This method calculates framewise displacement relative to the first volume and thresholds the displacement in order to classify outliers. From the included data, the framewise displacement analysis showed roughly 11% of participants had frames with a framewise displacement of more than 4 mm (the largest voxel size in the dataset), while roughly 30% of participants had frames with a framewise displacement of more than 1.5 mm (the smallest voxel size in the dataset) (Fig. 3a, Supplementary Table S2). In addition, analysis of outliers based on motion showed that 108 participants had more than 5% of their functional volumes marked as outliers (Fig. 3b, Supplementary Table S2). Increased head motion is expected in fMRI from patient groups and in particular children (see Supplementary Figure S3). Data of young participants may require extra processing steps, such as motion scrubbing^45,46^, Volterra expansion for general linear models^47^, and independent component analysis for artifact removal^48^. The complete list of framewise displacement values per subject and task can be found in Supplementary Figure S3 and Supplementary Table S3 (excel file).

### Correlated activation maps

The scans were grouped into four different categories for each hemisphere: hand movements and somatosensory stimulations from block designs (Motor2Class(Kids), Motor3Class or Sensory2Class), foot movements and somatosensory stimulations from block designs (Motor2Class and Sensory2Class), mouth movements from block designs (Motor2Class), and hand movements from event-related designs (Mapping3Fingers and Mapping5Fingers). Activation maps per scan were computed using a first-level analysis in SPM, by fitting the task design with the fMRI activity. For each scan in each group, a mean activation map of all scans from other participants in the same group was calculated^49^. The activation map of each scan was then correlated with the mean activation map of the group excluding the current scan. For scans with a correlation with the mean t-map lower than 0.3, the flat surface representation of the t-activation map was visually inspected. Inspected data were rated based on the level of noise, visually identified as small clusters of t-value activation strewn across the cortex, and the presence and size of clusters of activation in the SMC. Data with fewer clusters and more focal activity in the SMC was deemed good, while data with many dispersed smaller clusters and without an activation in the SMC was deemed bad. Only scans with task-related activations based on the correlation or visual inspection were kept in the dataset (see for example Fig. 3d).

### Response to the task

For illustration purposes, we performed a basic group-level analysis of the response to the Motor2Class using ‘fingers’ (fingertapping) paradigm, which is the most common task and paradigm in the dataset. A first-level analysis in SPM was conducted, where a general linear model was fitted to the fMRI data using the block design boxcar function (Fig. 3c). Then, we computed a second-level group statistic in SPM. Results of this analysis overlayed on a surface representation of the ‘fsaverage’ surface showed a clear activation over the left- and right-hand region of the sensorimotor cortex (Fig. 3d).

## Usage Notes

Below we summarise several participant and scan considerations to keep in mind when working with this dataset:

- Slice timing information is not available for the fMRI scans.
- For hand-related tasks, handedness and the hand used during the experiment did not coincide in 12.1% (N = 19) participants.
- The Mapping3Fingers and Mapping5Fingers tasks (participants: 21, 22, 23, 25, 27, 31, 37, 38 and 67), did not include whole-brain scans, which is reflected in the quality metrics. This indicates that special care needs to be taken when dealing with these scans, to ensure that no voxels outside of the scanning area are included in the analysis. Several other participants who performed the Motor2Class (subject 1, 7, 15, 16, 37 and 57) also did not have whole-brain scan but the field-of-view covered most of the sensorimotor cortex.
- In the Mapping3Fingers task (participants: 25, 27, 31, and 67), fMRI images consist of both magnitude and phase data, as described in the related *.json file. Particular attention must be paid to these scans to ensure that the phase images are not inadvertently used.
- For subject 62, session 3 T, the “imagine” condition from the Motor3Class task was used for movement of the other hand instead of imagining movement. The movement condition in these files is for right hand movement while the imagine condition is for left hand movement.
- The list of participants and scans with more than 10% motion outlier frames for all their scans are listed in Table 1.
- The list of participants and scans with a higher framewise displacement than 4 mm (the largest voxel size) for all their scans are listed in Table 2.

**Table 1:** Participants and scans with more than 10% motion outliers.

SUBJECT-ID	SESSION	TASK	RUN
7	7 T	Motor2Class	1
13	3 T	Sensory2Class	1
13	3 T	Motor2Class	2
15	1.5 T	Motor2Class	1
17	3 T	Motor2Class	1
21	7 T	Mapping5Fingers	1
30	3 T	Motor2Class	3
42	3 T	Motor2Class	1
46	3 T	Motor2Class	1
63	3 T	Motor2ClassKids	1
63	3 T	Motor2Class	1
64	3 T	Motor2ClassKids	1
70	3 T	Motor2ClassKids	1
85	3 T	Motor2Class	3
88	3 T	Motor2Class	2
93	3 T	Motor2Class	3
95	3 T	Motor2Class	4
96	3 T	Motor2Class	4
96	3 T	Motor2Class	1
98	3 T	Motor2Class	1
108	3 T	Motor2Class	3
108	3 T	Motor2Class	2
109	3 T	Motor2Class	3
116	3 T	Motor2Class	1
116	3 T	Motor2Class	2
120	3 T	Motor2Class	4
125	3 T	Motor2Class	3
126	3 T	Motor2Class	4
128	3 T	Motor2Class	4
129	3 T	Motor2Class	3
129	3 T	Motor2Class	1
129	3 T	Motor2Class	2
131	3 T	Motor2Class	4
136	3 T	Motor2Class	2
138	3 T	Motor2Class	1
142	3 T	Motor2Class	1
152	3 T	Motor2Class	1

**Table 2:** Participants and scans with framewise displacement larger than 4 mm.

SUBJECT-ID	SESSION	TASK	RUN
3	3 T	Motor2Class	1
3	3 T	Motor2ClassKids	1
7	7 T	Motor2Class	1
13	3 T	Sensory2Class	1
14	3 T	Motor2Class	1
15	1.5 T	Motor2Class	1
17	3 T	Motor2Class	1
25	3 T	Motor3Class	1
40	3 T	Motor2ClassKids	1
42	3 T	Motor2Class	1
46	3 T	Motor2Class	1
51	3 T	Motor2Class	1
62	3 T	Motor3Class	1
63	3 T	Motor2Class	1
63	3 T	Motor2ClassKids	1
70	3 T	Motor2Class	1
70	3 T	Motor2ClassKids	1
71	3 T	Motor2ClassKids	1
96	3 T	Motor2Class	4
109	3 T	Motor2Class	4

## Supplementary information

Supplementary Table S3

Supplementary Materials

## Data Availability

The code used to produce the validation metrics and generate preprocessing data are available in the public github repository: https://github.com/UMCU-RIBS/PANDA-fmri-dataset-validation. The ‘main’ function returns several tables containing framewise displacement metrics (function ‘fd_table’) and motion outliers (function ‘fsl_motion_outliers_table’). The functional scans should be aligned and coregistered with the anatomical T1 for each subject before running the ‘fd_table’ function. The function ‘fsl_motion_outliers_table’ requires FSL to be installed and can be used without preprocessing the data. All tables contain four columns that represent the subject number, session, task, and run. Additionally, the framewise displacement table contains an extra fifth column with framewise displacement between the current and preceding frame. Each row represents a frame in each run. The motion outliers table contains three additional columns, where the fifth column contains the number of frames, the sixth the number of motion outliers, and the seventh the percentage of frames categorised as motion outliers.
