# Supplementary material for: Large-scale fMRI dataset for the design of motor-based Brain-Computer Interfaces
Source: Sci Data. 2025 May 16;12:804. doi: 10.1038/s41597-025-05134-1 (PMC12084530; doi:10.1038/s41597-025-05134-1)

**Supplementary Materials**

**Supplementary Table S1 - Demographics.** Information about the subject’s ID number, age, sex (F=female, M=male), handedness (R=right hand, L=left hand), participant type (patient/healthy volunteer), type of movement performed (fingers=fingertapping or movement of multiple individual fingers; hand=open/close hand or hand palm; feet=both feet simultaneously; other body parts), the body side (R=right, L=left; only indicated once if all body parts where performed with the same side), as well as which body part was somatosensory stimulated (S).

| Sub-ID | Age | Sex | Handedness | Participant type | Hand | Other body parts |
| --- | --- | --- | --- | --- | --- | --- |
| 1 | 21 | M | R | Patient |  | mouth |
| 2 | 32 | F | R | Patient | L fingers | L foot |
| 3 | 7 | F | R | Healthy | R fingers, hand |  |
| 4 | 26 | M | R | Patient | R fingers, S-hand | tongue |
| 5 | 11 | M | R | Healthy | R fingers, hand |  |
| 6 | 29 | M | L | Patient | L fingers |  |
| 7 | 45 | M | R | Patient |  | tongue |
| 8 | 8 | M | R | Healthy | R fingers, hand |  |
| 9 | 17 | F | R | Patient | R fingers | tongue |
| 10 | 30 | F | R | Patient | R thumb finger, little finger | tongue |
| 11 | 9 | F | R | Healthy | R fingers, hand |  |
| 12 | 22 | F | R | Patient |  | tongue |
| 13 | 49 | M | L | Patient | R thumb finger, S-hand | tongue |
| 14 | 7 | M | R | Healthy | R fingers, hand |  |
| 15 | 15 | F | R | Patient | R thumb finger |  |
| 16 | 35 | M | L | Patient |  | mouth |
| 17 | 7 | F | R | Healthy | R fingers, hand |  |
| 18 | 18 | M | R | Patient | R thumb finger, index finger, little finger | R foot |
| 19 | 14 | F | R | Patient | R fingers |  |
| 20 | 15 | F | R | Patient | R fingers |  |
| 21 | 38 | F | L | Patient | R fingers |  |
| 22 | 42 | F | R | Patient | R fingers | tongue |
| 23 | 47 | F | R | Patient | R fingers | R foot |
| 24 | 18 | F | R | Patient | L thumb finger, index finger, little finger, fingers | tongue |
| 25 | 19 | F | R | Patient | R fingers |  |
| 26 | 51 | F | R | Patient |  | tongue |
| 27 | 29 | M | R | Patient | R fingers |  |
| 28 | 14 | F | R | Patient | L fingers | S-L foot |
| 29 | 27 | F | R | Patient | R thumb finger | tongue |
| 30 | 13 | M | R | Patient | R fingers, S-hand | arm, R foot |
| 31 | 42 | M | R | Patient | R fingers | tongue |
| 32 | 28 | M | R | Patient | R fingers |  |
| 33 | 21 | M | R | Patient | R fingers | R foot |
| 34 | 33 | F | R | Patient | R fingers |  |
| 35 | 20 | M | A | Patient | L thumb finger, index finger, little finger |  |
| 36 | 21 | M | R | Patient | L thumb finger, index finger | L foot |
| 37 | 45 | M | L | Patient | R fingers |  |
| 38 | 31 | F | R | Patient | L fingers |  |
| 39 | 17 | F | A | Patient | L thumb finger, little finger |  |
| 40 | 10 | F | R | Healthy | R fingers, hand |  |
| 41 | 10 | M | R | Healthy | R fingers, hand |  |
| 42 | 9 | M | R | Healthy | R fingers, hand |  |
| 43 | 16 | M | R | Patient |  | tongue |
| 44 | 36 | F | R | Patient |  | tongue |
| 45 | 26 | M | R | Patient | L fingers, S-hand | arm |
| 46 | 20 | M | R | Patient |  | tongue |
| 47 | 35 | F | R | Patient | R fingers |  |
| 48 | 26 | M | R | Patient |  | R foot |
| 49 | 12 | F | L | Patient | L fingers, S-hand | arm, S-L foot |
| 50 | 45 | M | R | Patient | R fingers | tongue |
| 51 | 9 | F | R | Healthy | R fingers, hand |  |
| 52 | 41 | F | L | Patient |  | tongue |
| 53 | 13 | F | L | Patient | R fingers | tongue |
| 54 | 18 | M | R | Patient | R fingers | tongue |
| 55 | 49 | M | R | Patient |  | tongue |
| 56 | 10 | M | L | Healthy | L fingers, hand |  |
| 57 | 58 | M | R | Patient |  | mouth |
| 58 | 18 | M | R | Patient | L fingers | tongue |
| 59 | 23 | M | R | Patient | L thumb finger, index finger, little finger, fingers, S-hand | tongue |
| 60 | 49 | M | R | Patient | R thumb finger, little finger, index finger |  |
| 61 | 33 | F | R | Patient | R thumb finger, index finger, fingers, S-hand | tongue |
| 62 | 21 | M | R | Patient | L fingers | R foot |
| 63 | 9 | F | R | Healthy | R fingers, hand |  |
| 64 | 7 | F | R | Healthy | R fingers, hand |  |
| 65 | 30 | F | L | Patient | R fingers |  |
| 66 | 50 | F | R | Patient | R fingers |  |
| 67 | 19 | M | R | Patient | R fingers |  |
| 68 | 28 | M | L | Patient | L fingers |  |
| 69 | 22 | M | A | Patient | R fingers, S-hand | tongue |
| 70 | 8 | M | L | Patient | R fingers, hand |  |
| 71 | 8 | F | R | Healthy | R fingers, hand |  |
| 72 | 11 | M | R | Patient | R fingers |  |
| 73 | 12 | M | R | Patient | L thumb finger, index finger, little finger, fingers, S-hand |  |
| 74 | 38 | F | R | Patient | L thumb finger |  |
| 75 | 6 | F | R | Healthy | R fingers, hand |  |
| 76 | 25 | M | R | Patient | R fingers |  |
| 77 | 14 | M | R | Patient | L fingers | tongue |
| 78 | 8 | F | L | Healthy | L fingers, hand |  |
| 79 | 38 | M | R | Healthy | L+R fingers | tongue, feet |
| 80 | 89 | F | R | Healthy | L+R fingers | tongue, feet |
| 81 | 76 | F | R | Healthy | L+R fingers | tongue, feet |
| 82 | 86 | F | R | Healthy | L+R fingers | tongue, feet |
| 83 | 25 | M | R | Healthy | L+R fingers | tongue, feet |
| 84 | 20 | M | R | Healthy | L+R fingers | tongue, feet |
| 85 | 20 | F | R | Healthy | L+R fingers | tongue, feet |
| 86 | 69 | M | R | Healthy | L+R fingers | tongue, feet |
| 87 | 46 | F | R | Healthy | L+R fingers | tongue, feet |
| 88 | 55 | F | R | Healthy | L+R fingers | tongue, feet |
| 89 | 57 | F | R | Healthy | L+R fingers | tongue, feet |
| 90 | 21 | F | R | Healthy | L+R fingers | tongue, feet |
| 91 | 18 | M | R | Healthy | L+R fingers | tongue, feet |
| 92 | 22 | F | R | Healthy | L+R fingers | tongue, feet |
| 93 | 70 | M | R | Healthy | L+R fingers | tongue, feet |
| 94 | 28 | F | R | Healthy | L+R fingers | tongue, feet |
| 95 | 18 | M | R | Healthy | L+R fingers | tongue, feet |
| 96 | 54 | F | R | Healthy | L+R fingers | tongue, feet |
| 97 | 67 | F | R | Healthy | L+R fingers | tongue, feet |
| 98 | 62 | M | R | Healthy | L+R fingers | tongue, feet |
| 99 | 79 | F | R | Healthy | L+R fingers | tongue, feet |
| 100 | 69 | F | R | Healthy | L+R fingers | tongue, feet |
| 101 | 29 | F | R | Healthy | L+R fingers | tongue, feet |
| 102 | 28 | F | R | Healthy | L+R fingers | tongue, feet |
| 103 | 28 | M | R | Healthy | L+R fingers | tongue, feet |
| 104 | 29 | F | R | Healthy | L+R fingers | tongue, feet |
| 105 | 19 | M | R | Healthy | L+R fingers | tongue, feet |
| 106 | 34 | M | R | Healthy | L+R fingers | tongue, feet |
| 107 | 46 | M | R | Healthy | L+R fingers | tongue, feet |
| 108 | 31 | M | R | Healthy | L+R fingers | tongue, feet |
| 109 | 65 | F | R | Healthy | L+R fingers | tongue, feet |
| 110 | 38 | M | R | Healthy | L+R fingers | tongue, feet |
| 111 | 30 | F | R | Healthy | L+R fingers | tongue, feet |
| 112 | 51 | M | R | Healthy | L+R fingers | tongue, feet |
| 113 | 67 | M | R | Healthy | L+R fingers | tongue, feet |
| 114 | 69 | M | R | Healthy | L+R fingers | tongue, feet |
| 115 | 63 | F | R | Healthy | L+R fingers | tongue, feet |
| 116 | 26 | F | R | Healthy | L+R fingers | tongue, feet |
| 117 | 40 | F | R | Healthy | L+R fingers | tongue, feet |
| 118 | 20 | F | R | Healthy | L+R fingers | tongue, feet |
| 119 | 46 | M | R | Healthy | L+R fingers | tongue, feet |
| 120 | 19 | M | R | Healthy | L+R fingers | tongue, feet |
| 121 | 70 | F | R | Healthy | L+R fingers | tongue, feet |
| 122 | 54 | F | R | Healthy | L+R fingers | tongue, feet |
| 123 | 72 | M | R | Healthy | L+R fingers | tongue, feet |
| 124 | 25 | M | R | Healthy | L+R fingers | tongue, feet |
| 125 | 74 | M | R | Healthy | L+R fingers | tongue, feet |
| 126 | 74 | F | R | Healthy | L+R fingers | tongue, feet |
| 127 | 44 | M | R | Healthy | L+R fingers | tongue, feet |
| 128 | 30 | F | R | Healthy | L+R fingers | tongue, feet |
| 129 | 68 | M | R | Healthy | L+R fingers | tongue, feet |
| 130 | 77 | M | R | Healthy | L+R fingers | tongue, feet |
| 131 | 22 | M | R | Healthy | L+R fingers | tongue, feet |
| 132 | 44 | M | R | Healthy | L+R fingers | tongue, feet |
| 133 | 22 | F | R | Healthy | L+R fingers | tongue, feet |
| 134 | 53 | F | R | Healthy | L+R fingers | tongue, feet |
| 135 | 47 | F | R | Healthy | L+R fingers | tongue, feet |
| 136 | 80 | M | R | Healthy | L+R fingers | tongue, feet |
| 137 | 58 | M | R | Healthy | L+R fingers | tongue, feet |
| 138 | 70 | F | R | Healthy | L+R fingers | tongue, feet |
| 139 | 86 | F | R | Healthy | L+R fingers | tongue, feet |
| 140 | 42 | F | R | Healthy | L+R fingers | tongue, feet |
| 141 | 77 | F | R | Healthy | L+R fingers | tongue, feet |
| 142 | 32 | M | R | Healthy | L+R fingers | tongue, feet |
| 143 | 36 | F | R | Healthy | L+R fingers | tongue, feet |
| 144 | 67 | M | R | Healthy | L+R fingers | tongue, feet |
| 145 | 44 | F | R | Healthy | L+R fingers | tongue, feet |
| 146 | 51 | F | R | Healthy | L+R fingers | tongue, feet |
| 147 | 38 | F | R | Healthy | L+R fingers | tongue, feet |
| 148 | 44 | M | R | Healthy | L+R fingers | tongue, feet |
| 149 | 72 | F | R | Healthy | L+R fingers | tongue, feet |
| 150 | 19 | F | R | Healthy | L+R fingers | tongue, feet |
| 151 | 24 | F | R | Healthy | L+R fingers | tongue, feet |
| 152 | 69 | M | R | Healthy | L+R fingers | tongue, feet |
| 153 | 28 | M | R | Healthy | L+R fingers | tongue, feet |
| 154 | 56 | M | R | Healthy | L+R fingers | tongue, feet |
| 155 | 16 | M | R | Patient | L+R fingers |  |

**Supplementary Table S2 – Quality metrics.** Framewise displacement and motion outliers per subject, task and run.

| Subject_ID | Session | Task | Run | Highest_Framewise_Displacement | Percentage_FSL_Motion_Outliers |
| --- | --- | --- | --- | --- | --- |
| 1 | 7T | Motor2Class | 1 | 0.20397527 | 0 |
| 2 | 1.5T | Motor2Class | 2 | 0.72348285 | 4.11764706 |
| 2 | 1.5T | Motor2Class | 1 | 0.70318667 | 2.94117647 |
| 3 | 3T | Motor2Class | 1 | 4.55243794 | 0 |
| 3 | 3T | Motor2ClassKids | 1 | 6.60186332 | 0 |
| 4 | 3T | Motor2Class | 1 | 0.68282232 | 4.07239819 |
| 4 | 3T | Sensory2Class | 1 | 0.38026096 | 2.94117647 |
| 4 | 3T | Motor3Class | 1 | 0.65854493 | 1.72215844 |
| 5 | 3T | Motor2ClassKids | 1 | 0.98039661 | 4.31818182 |
| 5 | 3T | Motor2Class | 1 | 0.84858239 | 4.07239819 |
| 6 | 3T | Motor2Class | 1 | 1.21429464 | 8.96551724 |
| 7 | 7T | Motor2Class | 1 | 5.3516465 | 13.372093 |
| 8 | 3T | Motor2ClassKids | 1 | 0.86976744 | 4.26966292 |
| 8 | 3T | Motor2Class | 1 | 0.60728195 | 3.39366516 |
| 9 | 3T | Motor2Class | 1 | 0.93274736 | 0 |
| 9 | 3T | Motor3Class | 1 | 1.13280721 | 0 |
| 10 | 1.5T | Motor2Class | 3 | 0.38432691 | 7.35294118 |
| 10 | 1.5T | Motor2Class | 1 | 0.48430748 | 3.52941176 |
| 10 | 1.5T | Motor2Class | 2 | 0.4333175 | 3.52941176 |
| 11 | 3T | Motor2ClassKids | 1 | 2.3642371 | 9.11111111 |
| 11 | 3T | Motor2Class | 1 | 3.59918668 | 7.91855204 |
| 12 | 3T | Motor2Class | 1 | 0.36187151 | 2.94117647 |
| 13 | 3T | Sensory2Class | 1 | 4.72022663 | 11.7647059 |
| 13 | 3T | Motor2Class | 2 | 0.79693604 | 11.3122172 |
| 13 | 3T | Motor2Class | 1 | 1.11586356 | 6.56108597 |
| 14 | 3T | Motor2Class | 1 | 5.76417279 | 9.2760181 |
| 14 | 3T | Motor2ClassKids | 1 | 0.88550435 | 9.04977376 |
| 15 | 1.5T | Motor2Class | 1 | 17.7542961 | 14.4117647 |
| 16 | 7T | Motor2Class | 1 | 0.34267851 | 7.55813953 |
| 17 | 3T | Motor2Class | 1 | 4.60194933 | 11.7647059 |
| 17 | 3T | Motor2ClassKids | 1 | 0.66977551 | 3.84615385 |
| 18 | 1.5T | Motor2Class | 1 | 0.16123687 | 3.82352941 |
| 18 | 1.5T2 | Motor2Class | 2 | 0.11384708 | 2.94117647 |
| 18 | 1.5T | Motor2Class | 2 | 0.10957845 | 1.76470588 |
| 18 | 1.5T2 | Motor2Class | 1 | 0.13626195 | 0.88235294 |
| 19 | 3T | Motor2Class | 1 | 2.42774525 | 8.27586207 |
| 20 | 3T | Motor2Class | 1 | 0.40660124 | 1.80995475 |
| 21 | 7T | Mapping5Fingers | 1 | 2.57833121 | 10.1156069 |
| 21 | 7T | Mapping5Fingers | 2 | 0.68130646 | 0.79710145 |
| 22 | 7T | Mapping5Fingers | 1 | 0.24961623 | 6.08695652 |
| 22 | 3T | Motor2Class | 2 | 0.54712575 | 3.39366516 |
| 22 | 3T | Motor2Class | 1 | 0.59517391 | 1.13122172 |
| 23 | 7T | Mapping5Fingers | 1 | 2.54297071 | 0 |
| 23 | 3T | Motor2Class | 1 | 0.43400524 | 2.26244344 |
| 23 | 3T | Motor2Class | 2 | 0.73217602 | 0.90497738 |
| 24 | 3T | Motor2Class | 2 | 0.5283775 | 4.29864253 |
| 24 | 3T | Motor3Class | 1 | 0.76002067 | 3.44431688 |
| 24 | 3T | Motor2Class | 1 | 0.34979553 | 3.39366516 |
| 24 | 3T | Motor2Class | 4 | 0.63599221 | 3.16742081 |
| 24 | 3T | Motor2Class | 3 | 0.35675603 | 2.71493213 |
| 25 | 3T | Motor2Class | 1 | 1.36955778 | 9.50226244 |
| 25 | 3T | Motor3Class | 1 | 7.06235282 | 8.61079219 |
| 25 | 7T | Mapping3Fingers | 2 | 0.32935322 | 7.8021978 |
| 25 | 7T | Mapping3Fingers | 1 | 0.51034867 | 5.27472527 |
| 26 | 3T | Motor2Class | 1 | 1.51440655 | 6.89655172 |
| 27 | 7T | Mapping3Fingers | 2 | 0.16651167 | 9.01098901 |
| 27 | 7T | Mapping3Fingers | 1 | 0.18953486 | 7.8021978 |
| 28 | 3T | Motor2Class | 2 | 0.49871601 | 5.20361991 |
| 28 | 3T | Motor2Class | 1 | 0.32360483 | 2.94117647 |
| 28 | 3T | Sensory2Class | 1 | 0.38488841 | 0.45248869 |
| 29 | 1.5T | Motor2Class | 1 | 0.32219988 | 6.76470588 |
| 29 | 1.5T | Motor2Class | 2 | 0.3111369 | 4.11764706 |
| 30 | 3T | Motor2Class | 3 | 0.86665441 | 10.8597285 |
| 30 | 3T | Sensory2Class | 1 | 0.64429272 | 8.59728507 |
| 30 | 3T | Motor2Class | 2 | 1.09778923 | 7.91855204 |
| 30 | 3T | Motor2Class | 1 | 0.83542211 | 6.10859729 |
| 31 | 3T | Motor2Class | 1 | 0.49066551 | 4.52488688 |
| 31 | 7T | Mapping3Fingers | 1 | 0.28044633 | 4.50549451 |
| 31 | 3T | Motor3Class | 1 | 0.37358668 | 0.9184845 |
| 32 | 3T | Motor2Class | 1 | 0.83137893 | 3.55648536 |
| 32 | 3T | Motor3Class | 1 | 1.16059766 | 2.06659013 |
| 33 | 3T | Motor2Class | 1 | 0.30600784 | 5.6561086 |
| 33 | 3T | Motor2Class | 2 | 0.30401491 | 0.90497738 |
| 34 | 3T | Motor3Class | 1 | 1.79956054 | 4.82204363 |
| 35 | 1.5T | Motor2Class | 2 | 0.19588571 | 2.64705882 |
| 35 | 1.5T | Motor2Class | 1 | 0.23136315 | 0.88235294 |
| 35 | 1.5T | Motor2Class | 3 | 0.25541461 | 0.88235294 |
| 36 | 1.5T | Motor2Class | 3 | 2.23907845 | 3.82352941 |
| 36 | 1.5T | Motor2Class | 2 | 0.82432871 | 3.52941176 |
| 36 | 1.5T | Motor2Class | 1 | 1.26474901 | 1.76470588 |
| 37 | 7T | Mapping5Fingers | 1 | 0.53773081 | 0 |
| 37 | 7T | Motor2Class | 1 | 0.51845659 | 0 |
| 38 | 7T | Mapping5Fingers | 1 | 0.49779904 | 0.86956522 |
| 39 | 1.5T | Motor2Class | 2 | 0.3626999 | 7.64705882 |
| 39 | 1.5T | Motor2Class | 1 | 0.26639185 | 4.70588235 |
| 40 | 3T | Motor2ClassKids | 1 | 11.7577675 | 8.88888889 |
| 40 | 3T | Motor2Class | 1 | 2.00740807 | 7.239819 |
| 41 | 3T | Motor2Class | 1 | 0.94525721 | 7.91855204 |
| 41 | 3T | Motor2ClassKids | 1 | 1.03462272 | 5.39325843 |
| 42 | 3T | Motor2Class | 1 | 4.60816 | 10.4072398 |
| 42 | 3T | Motor2ClassKids | 1 | 3.15060888 | 3.6199095 |
| 43 | 3T | Motor2Class | 1 | 1.22638767 | 5.42986425 |
| 44 | 3T | Motor2Class | 1 | 2.58696124 | 6.20689655 |
| 45 | 3T | Motor2Class | 3 | 0.51412919 | 2.94117647 |
| 45 | 3T | Sensory2Class | 1 | 0.57601742 | 1.80995475 |
| 45 | 3T | Motor2Class | 2 | 0.48591719 | 1.35746606 |
| 45 | 3T | Motor2Class | 1 | 0.45237277 | 0.90497738 |
| 46 | 3T | Motor2Class | 1 | 7.12355991 | 13.1221719 |
| 47 | 3T | Motor3Class | 1 | 0.83700181 | 4.24799082 |
| 48 | 3T | Motor2Class | 1 | 2.58087182 | 3.117506 |
| 49 | 3T | Motor2Class | 3 | 1.55227098 | 4.97737557 |
| 49 | 3T | Sensory2Class | 1 | 0.38033229 | 3.84615385 |
| 49 | 3T | Motor2Class | 2 | 0.6381956 | 3.39366516 |
| 49 | 3T | Motor2Class | 1 | 0.56344727 | 3.16742081 |
| 49 | 3T | Sensory2Class | 2 | 0.49394109 | 2.26244344 |
| 50 | 3T | Motor2Class | 1 | 0.30209159 | 0.67873303 |
| 50 | 3T | Motor2Class | 2 | 0.39155692 | 0.45248869 |
| 51 | 3T | Motor2ClassKids | 1 | 3.31327823 | 5.6561086 |
| 51 | 3T | Motor2Class | 1 | 4.57035457 | 4.52488688 |
| 52 | 3T | Motor2Class | 1 | 0.5717682 | 2.48868778 |
| 53 | 3T | Motor2Class | 2 | 0.79245199 | 5.6561086 |
| 53 | 3T | Motor2Class | 1 | 0.55852157 | 4.30839002 |
| 54 | 3T | Motor3Class | 1 | 1.95658981 | 2.87026406 |
| 54 | 3T | Motor2Class | 1 | 0.99405712 | 1.58371041 |
| 55 | 3T | Motor2Class | 1 | 2.05726565 | 6.78733032 |
| 56 | 3T | Motor2ClassKids | 1 | 0.99842104 | 8.82352941 |
| 56 | 3T | Motor2Class | 1 | 3.89952149 | 8.37104072 |
| 57 | 7T | Motor2Class | 1 | 1.09421847 | 6.97674419 |
| 58 | 3T | Motor2Class | 1 | 0.28127948 | 8.82352941 |
| 58 | 3T | Motor2Class | 2 | 0.62141522 | 7.69230769 |
| 59 | 3T | Motor2Class | 4 | 0.78686311 | 5.88235294 |
| 59 | 3T | Motor2Class | 2 | 0.24728781 | 2.71493213 |
| 59 | 3T | Motor3Class | 1 | 0.59777906 | 2.18140069 |
| 59 | 3T | Sensory2Class | 1 | 0.23724484 | 2.0361991 |
| 59 | 3T | Motor2Class | 1 | 0.43865915 | 1.80995475 |
| 59 | 3T | Motor2Class | 3 | 0.43581436 | 1.35746606 |
| 60 | 3T | Motor2Class | 2 | 1.17018554 | 4.07239819 |
| 60 | 3T | Motor2Class | 3 | 1.25834013 | 3.6199095 |
| 60 | 3T | Motor2Class | 1 | 1.07842925 | 2.26244344 |
| 61 | 3T | Motor3Class | 1 | 2.27180485 | 6.88863375 |
| 61 | 3T | Motor2Class | 1 | 0.6662237 | 5.88235294 |
| 61 | 3T | Sensory2Class | 1 | 0.86651295 | 3.16742081 |
| 61 | 3T | Motor2Class | 2 | 0.35973619 | 2.71493213 |
| 61 | 3T | Motor2Class | 3 | 0.51493336 | 1.13122172 |
| 62 | 3T | Motor3Class | 2 | 1.72229836 | 6.65901263 |
| 62 | 3T | Motor3Class | 1 | 5.42928371 | 5.97014925 |
| 63 | 3T | Motor2ClassKids | 1 | 11.5127846 | 16.2895928 |
| 63 | 3T | Motor2Class | 1 | 9.04282495 | 12.4434389 |
| 64 | 3T | Motor2ClassKids | 1 | 1.6862041 | 11.3122172 |
| 64 | 3T | Motor2Class | 1 | 0.52526123 | 5.42986425 |
| 65 | 3T | Motor2Class | 1 | 0.8379732 | 3.84615385 |
| 66 | 3T | Motor2Class | 1 | 0.96714941 | 0 |
| 67 | 7T | Mapping3Fingers | 2 | 0.42660638 | 5.93406593 |
| 67 | 7T | Mapping3Fingers | 1 | 0.10052617 | 5.6043956 |
| 67 | 3T | Motor3Class | 1 | 0.37797988 | 1.49253731 |
| 68 | 3T | Motor2Class | 1 | 0.44721003 | 2.48868778 |
| 69 | 3T | Motor2Class | 2 | 1.90752909 | 7.69230769 |
| 69 | 3T | Motor2Class | 1 | 0.69485345 | 4.75113122 |
| 69 | 3T | Sensory2Class | 1 | 0.54369708 | 2.26244344 |
| 70 | 3T | Motor2ClassKids | 1 | 7.32433575 | 11.3122172 |
| 70 | 3T | Motor2Class | 1 | 14.6827022 | 7.91855204 |
| 71 | 3T | Motor2ClassKids | 1 | 5.07664011 | 8.59728507 |
| 71 | 3T | Motor2Class | 1 | 1.99224871 | 6.33484163 |
| 72 | 3T | Motor2Class | 1 | 1.79261168 | 3.39366516 |
| 73 | 3T | Motor2Class | 1 | 0.46952769 | 6.10859729 |
| 73 | 3T | Motor3Class | 1 | 0.72734064 | 3.21469575 |
| 73 | 3T | Motor2Class | 2 | 0.40281843 | 2.94117647 |
| 73 | 3T | Sensory2Class | 1 | 0.74226272 | 2.94117647 |
| 73 | 3T | Motor2Class | 3 | 0.67140915 | 2.48868778 |
| 74 | 3T | Motor2Class | 1 | 0.39420082 | 0.66518847 |
| 75 | 3T | Motor2ClassKids | 1 | 2.48189632 | 8.59728507 |
| 75 | 3T | Motor2Class | 1 | 2.33528145 | 7.01357466 |
| 76 | 3T | Motor2Class | 1 | 0.61500803 | 5.51724138 |
| 77 | 3T | Motor2Class | 1 | 1.15304615 | 6.33484163 |
| 77 | 3T | Motor2Class | 2 | 0.58587518 | 1.58371041 |
| 78 | 3T | Motor2Class | 1 | 1.27630474 | 7.01357466 |
| 78 | 3T | Motor2ClassKids | 1 | 1.70436488 | 4.07239819 |
| 79 | 3T | Motor2Class | 2 | 0.62202632 | 0 |
| 79 | 3T | Motor2Class | 1 | 0.89800823 | 1.66666667 |
| 79 | 3T | Motor2Class | 3 | 0.76757323 | 1.66666667 |
| 79 | 3T | Motor2Class | 4 | 0.63293961 | 1.66666667 |
| 80 | 3T | Motor2Class | 4 | 0.46492965 | 9.16666667 |
| 80 | 3T | Motor2Class | 3 | 0.31339884 | 5 |
| 80 | 3T | Motor2Class | 1 | 0.34291447 | 1.66666667 |
| 80 | 3T | Motor2Class | 2 | 0.37133996 | 1.66666667 |
| 81 | 3T | Motor2Class | 4 | 0.47935819 | 2.5 |
| 81 | 3T | Motor2Class | 1 | 0.17039983 | 1.66666667 |
| 81 | 3T | Motor2Class | 2 | 0.13686821 | 1.66666667 |
| 81 | 3T | Motor2Class | 3 | 0.11891614 | 1.66666667 |
| 82 | 3T | Motor2Class | 1 | 0.78075542 | 8.33333333 |
| 82 | 3T | Motor2Class | 4 | 1.00657044 | 8.33333333 |
| 82 | 3T | Motor2Class | 2 | 0.66345852 | 5.83333333 |
| 82 | 3T | Motor2Class | 3 | 0.46320695 | 5 |
| 83 | 3T | Motor2Class | 2 | 0.50255884 | 6.66666667 |
| 83 | 3T | Motor2Class | 3 | 0.39804833 | 5.83333333 |
| 83 | 3T | Motor2Class | 1 | 0.45545505 | 5 |
| 83 | 3T | Motor2Class | 4 | 0.68924508 | 3.33333333 |
| 84 | 3T | Motor2Class | 2 | 0.47680294 | 7.5 |
| 84 | 3T | Motor2Class | 4 | 0.78838335 | 5.83333333 |
| 84 | 3T | Motor2Class | 1 | 0.30333576 | 4.16666667 |
| 84 | 3T | Motor2Class | 3 | 0.29127645 | 1.66666667 |
| 85 | 3T | Motor2Class | 3 | 1.01355575 | 11.6666667 |
| 85 | 3T | Motor2Class | 1 | 1.62829643 | 9.16666667 |
| 85 | 3T | Motor2Class | 2 | 1.96469702 | 9.16666667 |
| 85 | 3T | Motor2Class | 4 | 1.72205418 | 1.66666667 |
| 86 | 3T | Motor2Class | 2 | 0.2801219 | 4.16666667 |
| 86 | 3T | Motor2Class | 1 | 0.21619584 | 3.33333333 |
| 86 | 3T | Motor2Class | 4 | 0.68489964 | 2.5 |
| 86 | 3T | Motor2Class | 3 | 0.33019148 | 1.66666667 |
| 87 | 3T | Motor2Class | 3 | 0.43319954 | 8.33333333 |
| 87 | 3T | Motor2Class | 4 | 1.2598462 | 7.73809524 |
| 87 | 3T | Motor2Class | 1 | 0.34801182 | 7.14285714 |
| 87 | 3T | Motor2Class | 2 | 0.29995491 | 7.14285714 |
| 88 | 3T | Motor2Class | 2 | 2.08267967 | 13.3333333 |
| 88 | 3T | Motor2Class | 1 | 0.6440248 | 8.33333333 |
| 88 | 3T | Motor2Class | 3 | 0.45474869 | 6.66666667 |
| 88 | 3T | Motor2Class | 4 | 1.35097504 | 5 |
| 89 | 3T | Motor2Class | 4 | 0.33324204 | 8.33333333 |
| 89 | 3T | Motor2Class | 1 | 0.17525031 | 7.5 |
| 89 | 3T | Motor2Class | 2 | 0.26972738 | 3.33333333 |
| 89 | 3T | Motor2Class | 3 | 0.12981395 | 2.5 |
| 90 | 3T | Motor2Class | 4 | 0.37590967 | 4.76190476 |
| 90 | 3T | Motor2Class | 1 | 0.1388238 | 3.57142857 |
| 90 | 3T | Motor2Class | 2 | 0.0851017 | 3.57142857 |
| 90 | 3T | Motor2Class | 3 | 0.1453684 | 2.97619048 |
| 91 | 3T | Motor2Class | 2 | 0.23939534 | 5.35714286 |
| 91 | 3T | Motor2Class | 1 | 0.1234854 | 4.16666667 |
| 91 | 3T | Motor2Class | 3 | 0.25152611 | 4.16666667 |
| 91 | 3T | Motor2Class | 4 | 0.16424173 | 2.97619048 |
| 92 | 3T | Motor2Class | 4 | 0.25669121 | 5 |
| 92 | 3T | Motor2Class | 2 | 0.10416917 | 4.16666667 |
| 92 | 3T | Motor2Class | 3 | 0.17035088 | 4.16666667 |
| 92 | 3T | Motor2Class | 1 | 0.22100423 | 2.5 |
| 93 | 3T | Motor2Class | 3 | 0.68734022 | 10 |
| 93 | 3T | Motor2Class | 1 | 0.62452017 | 9.16666667 |
| 93 | 3T | Motor2Class | 4 | 1.19771883 | 9.16666667 |
| 93 | 3T | Motor2Class | 2 | 1.67397303 | 6.66666667 |
| 94 | 3T | Motor2Class | 4 | 0.66183886 | 5.83333333 |
| 94 | 3T | Motor2Class | 2 | 0.27025417 | 4.16666667 |
| 94 | 3T | Motor2Class | 3 | 0.70073908 | 3.33333333 |
| 94 | 3T | Motor2Class | 1 | 0.29850187 | 1.66666667 |
| 95 | 3T | Motor2Class | 4 | 0.73204126 | 11.9047619 |
| 95 | 3T | Motor2Class | 2 | 0.34146317 | 3.57142857 |
| 95 | 3T | Motor2Class | 3 | 0.28683519 | 3.57142857 |
| 95 | 3T | Motor2Class | 1 | 0.20256848 | 1.78571429 |
| 96 | 3T | Motor2Class | 4 | 5.69061919 | 12.5 |
| 96 | 3T | Motor2Class | 1 | 0.54486925 | 10.8333333 |
| 96 | 3T | Motor2Class | 3 | 0.78500129 | 7.5 |
| 96 | 3T | Motor2Class | 2 | 0.66876588 | 5.83333333 |
| 97 | 3T | Motor2Class | 1 | 0.21533154 | 7.5 |
| 97 | 3T | Motor2Class | 2 | 0.45294363 | 2.5 |
| 97 | 3T | Motor2Class | 3 | 0.3452024 | 2.5 |
| 97 | 3T | Motor2Class | 4 | 1.17033635 | 2.5 |
| 98 | 3T | Motor2Class | 1 | 1.33409118 | 13.3333333 |
| 98 | 3T | Motor2Class | 2 | 0.69136498 | 9.16666667 |
| 98 | 3T | Motor2Class | 3 | 1.26135692 | 7.5 |
| 98 | 3T | Motor2Class | 4 | 1.10225585 | 6.66666667 |
| 99 | 3T | Motor2Class | 1 | 0.67911534 | 9.16666667 |
| 99 | 3T | Motor2Class | 4 | 1.12345225 | 7.5 |
| 99 | 3T | Motor2Class | 2 | 0.99378982 | 5.83333333 |
| 99 | 3T | Motor2Class | 3 | 1.05410957 | 1.66666667 |
| 100 | 3T | Motor2Class | 1 | 0.58119465 | 5.83333333 |
| 100 | 3T | Motor2Class | 2 | 0.33585925 | 5 |
| 100 | 3T | Motor2Class | 4 | 0.40098839 | 3.33333333 |
| 100 | 3T | Motor2Class | 3 | 0.43528702 | 1.66666667 |
| 101 | 3T | Motor2Class | 1 | 0.13551626 | 2.97619048 |
| 101 | 3T | Motor2Class | 2 | 0.15869992 | 1.78571429 |
| 101 | 3T | Motor2Class | 4 | 0.1987445 | 1.78571429 |
| 101 | 3T | Motor2Class | 3 | 0.12822976 | 1.19047619 |
| 102 | 3T | Motor2Class | 2 | 0.09365352 | 5.35714286 |
| 102 | 3T | Motor2Class | 3 | 0.10576617 | 4.16666667 |
| 102 | 3T | Motor2Class | 1 | 0.11323539 | 2.97619048 |
| 102 | 3T | Motor2Class | 4 | 0.1411615 | 2.97619048 |
| 103 | 3T | Motor2Class | 1 | 0.55045902 | 7.5 |
| 103 | 3T | Motor2Class | 3 | 0.31968579 | 5 |
| 103 | 3T | Motor2Class | 4 | 1.48438713 | 5 |
| 103 | 3T | Motor2Class | 2 | 0.19760811 | 4.16666667 |
| 104 | 3T | Motor2Class | 4 | 0.34205338 | 4.16666667 |
| 104 | 3T | Motor2Class | 1 | 0.20821873 | 1.66666667 |
| 104 | 3T | Motor2Class | 2 | 0.14852508 | 1.66666667 |
| 104 | 3T | Motor2Class | 3 | 0.28452965 | 1.66666667 |
| 105 | 3T | Motor2Class | 1 | 0.25591448 | 5 |
| 105 | 3T | Motor2Class | 3 | 0.10868792 | 4.16666667 |
| 105 | 3T | Motor2Class | 4 | 0.29026594 | 4.16666667 |
| 105 | 3T | Motor2Class | 2 | 0.14545992 | 2.5 |
| 106 | 3T | Motor2Class | 3 | 3.07123218 | 8.33333333 |
| 106 | 3T | Motor2Class | 4 | 1.17067705 | 6.66666667 |
| 106 | 3T | Motor2Class | 1 | 0.177151 | 1.66666667 |
| 106 | 3T | Motor2Class | 2 | 0.28143978 | 1.66666667 |
| 107 | 3T | Motor2Class | 1 | 0.16170224 | 8.33333333 |
| 107 | 3T | Motor2Class | 3 | 0.22774252 | 8.33333333 |
| 107 | 3T | Motor2Class | 2 | 0.09799371 | 7.5 |
| 107 | 3T | Motor2Class | 4 | 0.3764164 | 7.5 |
| 108 | 3T | Motor2Class | 3 | 2.69565075 | 15 |
| 108 | 3T | Motor2Class | 2 | 1.72061456 | 10.8333333 |
| 108 | 3T | Motor2Class | 1 | 2.15658568 | 9.16666667 |
| 108 | 3T | Motor2Class | 4 | 2.18598611 | 5.83333333 |
| 109 | 3T | Motor2Class | 3 | 0.93234544 | 10 |
| 109 | 3T | Motor2Class | 2 | 1.61175579 | 7.5 |
| 109 | 3T | Motor2Class | 1 | 1.26577104 | 5 |
| 109 | 3T | Motor2Class | 4 | 7.40967671 | 5 |
| 110 | 3T | Motor2Class | 2 | 0.59352187 | 4.16666667 |
| 110 | 3T | Motor2Class | 3 | 0.59079046 | 3.33333333 |
| 110 | 3T | Motor2Class | 4 | 0.93136368 | 3.33333333 |
| 110 | 3T | Motor2Class | 1 | 0.26214091 | 2.5 |
| 111 | 3T | Motor2Class | 4 | 0.33519818 | 5.83333333 |
| 111 | 3T | Motor2Class | 2 | 0.18230943 | 2.5 |
| 111 | 3T | Motor2Class | 1 | 0.11457639 | 1.66666667 |
| 111 | 3T | Motor2Class | 3 | 0.12135331 | 1.66666667 |
| 112 | 3T | Motor2Class | 1 | 0.46611326 | 9.16666667 |
| 112 | 3T | Motor2Class | 3 | 0.36633798 | 6.66666667 |
| 112 | 3T | Motor2Class | 2 | 0.43972367 | 5.83333333 |
| 112 | 3T | Motor2Class | 4 | 0.5961407 | 5.83333333 |
| 113 | 3T | Motor2Class | 1 | 1.09047793 | 5 |
| 113 | 3T | Motor2Class | 4 | 1.41234441 | 3.33333333 |
| 113 | 3T | Motor2Class | 2 | 0.23589673 | 1.66666667 |
| 113 | 3T | Motor2Class | 3 | 0.31448981 | 1.66666667 |
| 114 | 3T | Motor2Class | 1 | 0.0937684 | 5 |
| 114 | 3T | Motor2Class | 2 | 0.1155927 | 4.16666667 |
| 114 | 3T | Motor2Class | 4 | 0.25351615 | 4.16666667 |
| 114 | 3T | Motor2Class | 3 | 0.09674265 | 2.5 |
| 115 | 3T | Motor2Class | 1 | 0.56776497 | 5.83333333 |
| 115 | 3T | Motor2Class | 4 | 1.99388575 | 5.83333333 |
| 115 | 3T | Motor2Class | 2 | 1.05593933 | 4.16666667 |
| 115 | 3T | Motor2Class | 3 | 0.66776033 | 2.5 |
| 116 | 3T | Motor2Class | 1 | 1.16549807 | 12.5 |
| 116 | 3T | Motor2Class | 2 | 0.59907261 | 10 |
| 116 | 3T | Motor2Class | 3 | 0.76407011 | 7.5 |
| 116 | 3T | Motor2Class | 4 | 1.86545996 | 7.5 |
| 117 | 3T | Motor2Class | 4 | 1.10562505 | 7.5 |
| 117 | 3T | Motor2Class | 2 | 0.2645126 | 4.16666667 |
| 117 | 3T | Motor2Class | 3 | 0.13316186 | 2.5 |
| 117 | 3T | Motor2Class | 1 | 0.18146102 | 1.66666667 |
| 118 | 3T | Motor2Class | 4 | 0.39450216 | 3.33333333 |
| 118 | 3T | Motor2Class | 1 | 0.08717761 | 1.66666667 |
| 118 | 3T | Motor2Class | 2 | 0.15674493 | 1.66666667 |
| 118 | 3T | Motor2Class | 3 | 0.07707956 | 1.66666667 |
| 119 | 3T | Motor2Class | 1 | 0.35142267 | 9.16666667 |
| 119 | 3T | Motor2Class | 4 | 0.45350852 | 6.66666667 |
| 119 | 3T | Motor2Class | 2 | 0.29671606 | 5 |
| 119 | 3T | Motor2Class | 3 | 0.24313099 | 2.5 |
| 120 | 3T | Motor2Class | 4 | 0.96240609 | 11.6666667 |
| 120 | 3T | Motor2Class | 2 | 0.35748786 | 4.16666667 |
| 120 | 3T | Motor2Class | 3 | 0.2374883 | 2.5 |
| 120 | 3T | Motor2Class | 1 | 0.35378824 | 1.66666667 |
| 121 | 3T | Motor2Class | 1 | 0.76086982 | 7.5 |
| 121 | 3T | Motor2Class | 4 | 0.49161317 | 6.66666667 |
| 121 | 3T | Motor2Class | 3 | 0.2185773 | 2.5 |
| 121 | 3T | Motor2Class | 2 | 0.26354014 | 1.66666667 |
| 122 | 3T | Motor2Class | 1 | 0.1864306 | 4.16666667 |
| 122 | 3T | Motor2Class | 2 | 0.21623703 | 4.16666667 |
| 122 | 3T | Motor2Class | 3 | 0.2958845 | 4.16666667 |
| 122 | 3T | Motor2Class | 4 | 0.59940143 | 2.5 |
| 123 | 3T | Motor2Class | 2 | 0.1700391 | 7.5 |
| 123 | 3T | Motor2Class | 4 | 0.65642931 | 5.83333333 |
| 123 | 3T | Motor2Class | 1 | 0.31171508 | 4.16666667 |
| 123 | 3T | Motor2Class | 3 | 0.14119052 | 1.66666667 |
| 124 | 3T | Motor2Class | 3 | 0.37113137 | 4.16666667 |
| 124 | 3T | Motor2Class | 1 | 0.88521674 | 3.33333333 |
| 124 | 3T | Motor2Class | 2 | 0.09038912 | 3.33333333 |
| 124 | 3T | Motor2Class | 4 | 0.56782754 | 1.66666667 |
| 125 | 3T | Motor2Class | 3 | 2.11614218 | 10.8333333 |
| 125 | 3T | Motor2Class | 1 | 0.83645064 | 8.33333333 |
| 125 | 3T | Motor2Class | 2 | 2.49131673 | 4.16666667 |
| 125 | 3T | Motor2Class | 4 | 1.15397832 | 3.33333333 |
| 126 | 3T | Motor2Class | 4 | 1.51249884 | 11.6666667 |
| 126 | 3T | Motor2Class | 1 | 0.53537468 | 4.16666667 |
| 126 | 3T | Motor2Class | 2 | 0.40011472 | 3.33333333 |
| 126 | 3T | Motor2Class | 3 | 0.64954088 | 3.33333333 |
| 127 | 3T | Motor2Class | 3 | 0.16503089 | 5 |
| 127 | 3T | Motor2Class | 1 | 0.33064992 | 4.16666667 |
| 127 | 3T | Motor2Class | 4 | 0.46432365 | 4.16666667 |
| 127 | 3T | Motor2Class | 2 | 0.13059151 | 1.66666667 |
| 128 | 3T | Motor2Class | 4 | 0.29174548 | 10 |
| 128 | 3T | Motor2Class | 2 | 0.14174461 | 5 |
| 128 | 3T | Motor2Class | 1 | 0.2333376 | 2.5 |
| 128 | 3T | Motor2Class | 3 | 0.19185329 | 2.5 |
| 129 | 3T | Motor2Class | 3 | 0.83878616 | 10.8333333 |
| 129 | 3T | Motor2Class | 1 | 0.51388916 | 10 |
| 129 | 3T | Motor2Class | 2 | 1.31916247 | 10 |
| 129 | 3T | Motor2Class | 4 | 1.19559507 | 7.5 |
| 130 | 3T | Motor2Class | 1 | 0.98060903 | 9.16666667 |
| 130 | 3T | Motor2Class | 3 | 0.59003505 | 8.33333333 |
| 130 | 3T | Motor2Class | 2 | 0.69773585 | 5.83333333 |
| 130 | 3T | Motor2Class | 4 | 1.10990858 | 3.33333333 |
| 131 | 3T | Motor2Class | 4 | 0.83584436 | 10 |
| 131 | 3T | Motor2Class | 3 | 0.11972952 | 9.16666667 |
| 131 | 3T | Motor2Class | 1 | 0.07958039 | 5 |
| 131 | 3T | Motor2Class | 2 | 0.0699496 | 3.33333333 |
| 132 | 3T | Motor2Class | 1 | 0.2559898 | 4.16666667 |
| 132 | 3T | Motor2Class | 2 | 0.04982665 | 1.66666667 |
| 132 | 3T | Motor2Class | 3 | 0.14150227 | 1.66666667 |
| 132 | 3T | Motor2Class | 4 | 0.23235245 | 1.66666667 |
| 133 | 3T | Motor2Class | 3 | 0.19927133 | 7.14285714 |
| 133 | 3T | Motor2Class | 2 | 0.27830992 | 4.16666667 |
| 133 | 3T | Motor2Class | 1 | 0.24782654 | 2.97619048 |
| 133 | 3T | Motor2Class | 4 | 0.2280776 | 2.97619048 |
| 134 | 3T | Motor2Class | 1 | 0.14129691 | 3.33333333 |
| 134 | 3T | Motor2Class | 4 | 0.18091607 | 2.5 |
| 134 | 3T | Motor2Class | 2 | 0.13045025 | 1.66666667 |
| 134 | 3T | Motor2Class | 3 | 0.12322625 | 1.66666667 |
| 135 | 3T | Motor2Class | 2 | 0.10880801 | 5 |
| 135 | 3T | Motor2Class | 4 | 0.31019677 | 5 |
| 135 | 3T | Motor2Class | 3 | 0.11431991 | 4.16666667 |
| 135 | 3T | Motor2Class | 1 | 0.08247348 | 2.5 |
| 136 | 3T | Motor2Class | 2 | 0.78052741 | 11.6666667 |
| 136 | 3T | Motor2Class | 1 | 1.16898297 | 6.66666667 |
| 136 | 3T | Motor2Class | 3 | 0.85259102 | 6.66666667 |
| 136 | 3T | Motor2Class | 4 | 1.32874458 | 4.16666667 |
| 137 | 3T | Motor2Class | 1 | 0.541224 | 6.66666667 |
| 137 | 3T | Motor2Class | 2 | 0.51896263 | 4.16666667 |
| 137 | 3T | Motor2Class | 3 | 0.56909612 | 4.16666667 |
| 137 | 3T | Motor2Class | 4 | 0.97599573 | 3.33333333 |
| 138 | 3T | Motor2Class | 1 | 0.68919629 | 10 |
| 138 | 3T | Motor2Class | 2 | 0.67486284 | 6.66666667 |
| 138 | 3T | Motor2Class | 3 | 0.56643005 | 5 |
| 138 | 3T | Motor2Class | 4 | 0.62898058 | 3.33333333 |
| 139 | 3T | Motor2Class | 4 | 1.44971844 | 5.83333333 |
| 139 | 3T | Motor2Class | 1 | 0.47576287 | 4.16666667 |
| 139 | 3T | Motor2Class | 2 | 0.36743316 | 4.16666667 |
| 139 | 3T | Motor2Class | 3 | 0.22720084 | 1.66666667 |
| 140 | 3T | Motor2Class | 4 | 0.38192117 | 8.33333333 |
| 140 | 3T | Motor2Class | 1 | 0.17463626 | 7.5 |
| 140 | 3T | Motor2Class | 2 | 0.15312096 | 3.33333333 |
| 140 | 3T | Motor2Class | 3 | 0.09876869 | 1.66666667 |
| 141 | 3T | Motor2Class | 3 | 1.1221509 | 7.5 |
| 141 | 3T | Motor2Class | 2 | 0.3907942 | 5 |
| 141 | 3T | Motor2Class | 1 | 1.57845216 | 4.16666667 |
| 141 | 3T | Motor2Class | 4 | 1.02540971 | 4.16666667 |
| 142 | 3T | Motor2Class | 1 | 0.54727934 | 13.3333333 |
| 142 | 3T | Motor2Class | 2 | 0.29168892 | 5.83333333 |
| 142 | 3T | Motor2Class | 3 | 0.30255131 | 3.33333333 |
| 142 | 3T | Motor2Class | 4 | 0.67058602 | 3.33333333 |
| 143 | 3T | Motor2Class | 1 | 0.13748188 | 3.33333333 |
| 143 | 3T | Motor2Class | 2 | 0.17746768 | 3.33333333 |
| 143 | 3T | Motor2Class | 4 | 0.2677861 | 3.33333333 |
| 143 | 3T | Motor2Class | 3 | 0.20497904 | 2.5 |
| 144 | 3T | Motor2Class | 4 | 0.23811417 | 5.83333333 |
| 144 | 3T | Motor2Class | 1 | 0.28712155 | 3.33333333 |
| 144 | 3T | Motor2Class | 3 | 0.28051331 | 3.33333333 |
| 144 | 3T | Motor2Class | 2 | 0.23528216 | 1.66666667 |
| 145 | 3T | Motor2Class | 1 | 0.42878137 | 7.5 |
| 145 | 3T | Motor2Class | 4 | 0.28288069 | 4.16666667 |
| 145 | 3T | Motor2Class | 2 | 0.2320884 | 3.33333333 |
| 145 | 3T | Motor2Class | 3 | 0.15713846 | 1.66666667 |
| 146 | 3T | Motor2Class | 2 | 0.23939534 | 5.35714286 |
| 146 | 3T | Motor2Class | 1 | 0.1234854 | 4.16666667 |
| 146 | 3T | Motor2Class | 3 | 0.25152611 | 4.16666667 |
| 146 | 3T | Motor2Class | 4 | 0.16424173 | 2.97619048 |
| 147 | 3T | Motor2Class | 3 | 0.08283226 | 5 |
| 147 | 3T | Motor2Class | 4 | 0.19596387 | 5 |
| 147 | 3T | Motor2Class | 1 | 0.35885151 | 3.33333333 |
| 147 | 3T | Motor2Class | 2 | 0.11960391 | 3.33333333 |
| 148 | 3T | Motor2Class | 2 | 0.2498165 | 9.16666667 |
| 148 | 3T | Motor2Class | 3 | 0.23734449 | 4.16666667 |
| 148 | 3T | Motor2Class | 4 | 0.24323474 | 4.16666667 |
| 148 | 3T | Motor2Class | 1 | 0.10429687 | 2.5 |
| 149 | 3T | Motor2Class | 1 | 0.21231876 | 2.5 |
| 149 | 3T | Motor2Class | 2 | 0.13418023 | 1.66666667 |
| 149 | 3T | Motor2Class | 3 | 0.37500211 | 1.66666667 |
| 149 | 3T | Motor2Class | 4 | 0.76301904 | 1.66666667 |
| 150 | 3T | Motor2Class | 1 | 0.31829099 | 5 |
| 150 | 3T | Motor2Class | 4 | 0.36270254 | 4.16666667 |
| 150 | 3T | Motor2Class | 2 | 0.37156775 | 2.5 |
| 150 | 3T | Motor2Class | 3 | 0.34074523 | 1.66666667 |
| 151 | 3T | Motor2Class | 3 | 0.88177647 | 5.83333333 |
| 151 | 3T | Motor2Class | 1 | 0.54277954 | 5 |
| 151 | 3T | Motor2Class | 2 | 0.21863072 | 4.16666667 |
| 151 | 3T | Motor2Class | 4 | 0.86656334 | 2.5 |
| 152 | 3T | Motor2Class | 1 | 0.79797729 | 11.6666667 |
| 152 | 3T | Motor2Class | 2 | 0.72068644 | 7.5 |
| 152 | 3T | Motor2Class | 3 | 0.62716964 | 3.33333333 |
| 152 | 3T | Motor2Class | 4 | 0.38942796 | 1.66666667 |
| 153 | 3T | Motor2Class | 4 | 0.67119433 | 6.66666667 |
| 153 | 3T | Motor2Class | 3 | 0.15937766 | 2.5 |
| 153 | 3T | Motor2Class | 1 | 0.17321918 | 1.66666667 |
| 153 | 3T | Motor2Class | 2 | 0.33520595 | 1.66666667 |
| 154 | 3T | Motor2Class | 2 | 0.4515271 | 5 |
| 154 | 3T | Motor2Class | 1 | 0.29099827 | 4.16666667 |
| 154 | 3T | Motor2Class | 3 | 0.4586885 | 4.16666667 |
| 154 | 3T | Motor2Class | 4 | 1.06068809 | 2.5 |
| 155 | 3T | Motor2Class | 1 | 0.5347576 | 2.17391304 |

**Supplementary Figure S1 – Brain coverage.** Probabilistic maps of fMRI brain coverage per scanner (1.5T, 3T and 7T, and all together). Color scale indicates the aggregate coverage from purple (1 scan) to red (all scans).


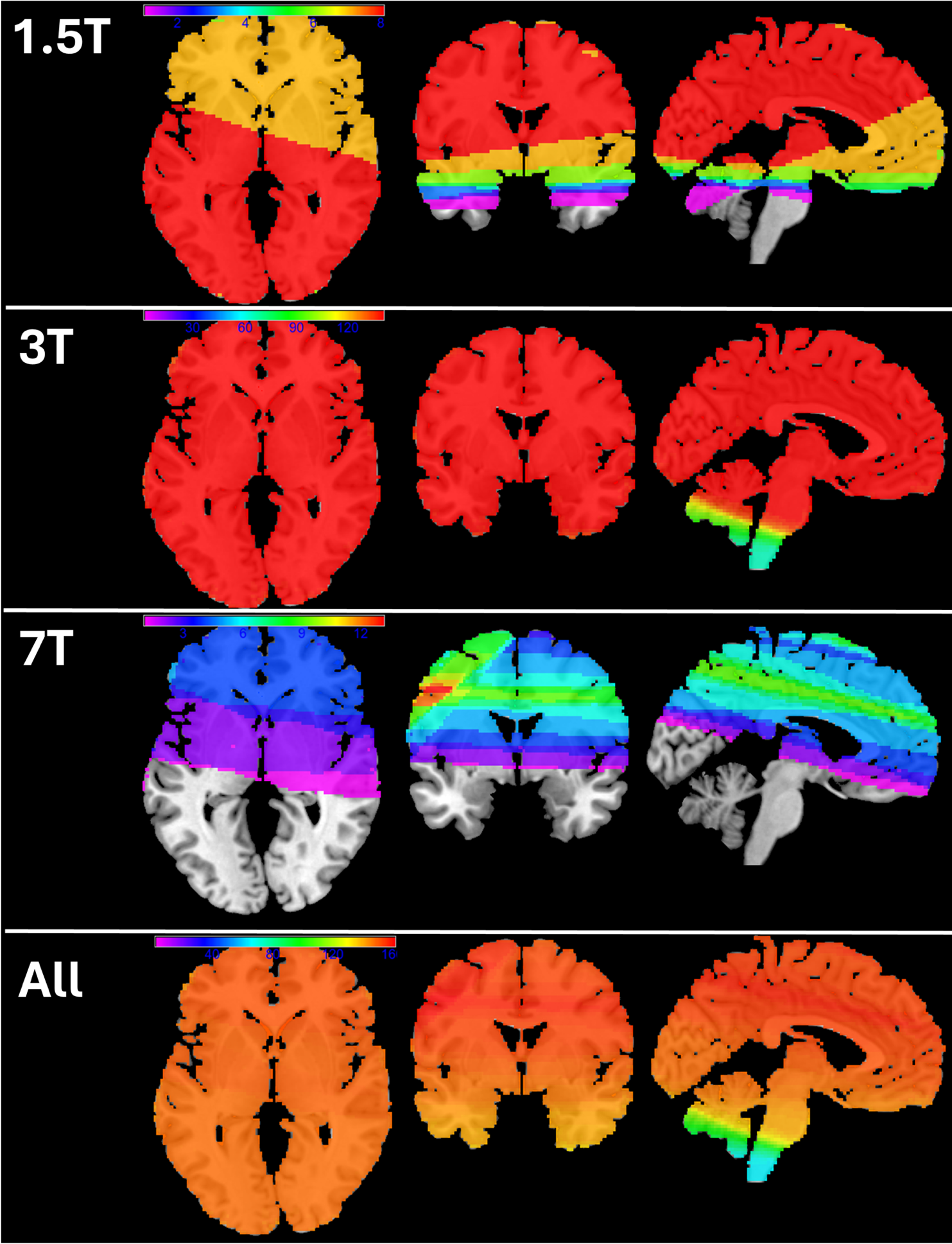


**Supplementary Figure S2 – Temporal signal-to-noise ratio.** Temporal signal-to-noise ratio (SNR) measures signal dropout and effects of noise over time. The temporal SNR was calculated for each voxel as the mean of the voxel time series divided by the standard deviation of the voxel time series. Temporal SNR was performed on realigned, resliced and high-pass filtered (100s cutoff) data. The average temporal SNR across participants was displayed on a normalized MNI volume space per scanner type (1.5, 3 and 7T). Values per participant (averaged across runs) can be computed using the “temporal_snr” function in the git repository <https://github.com/UMCU-RIBS/PANDA-fmri-dataset-validation>.

**
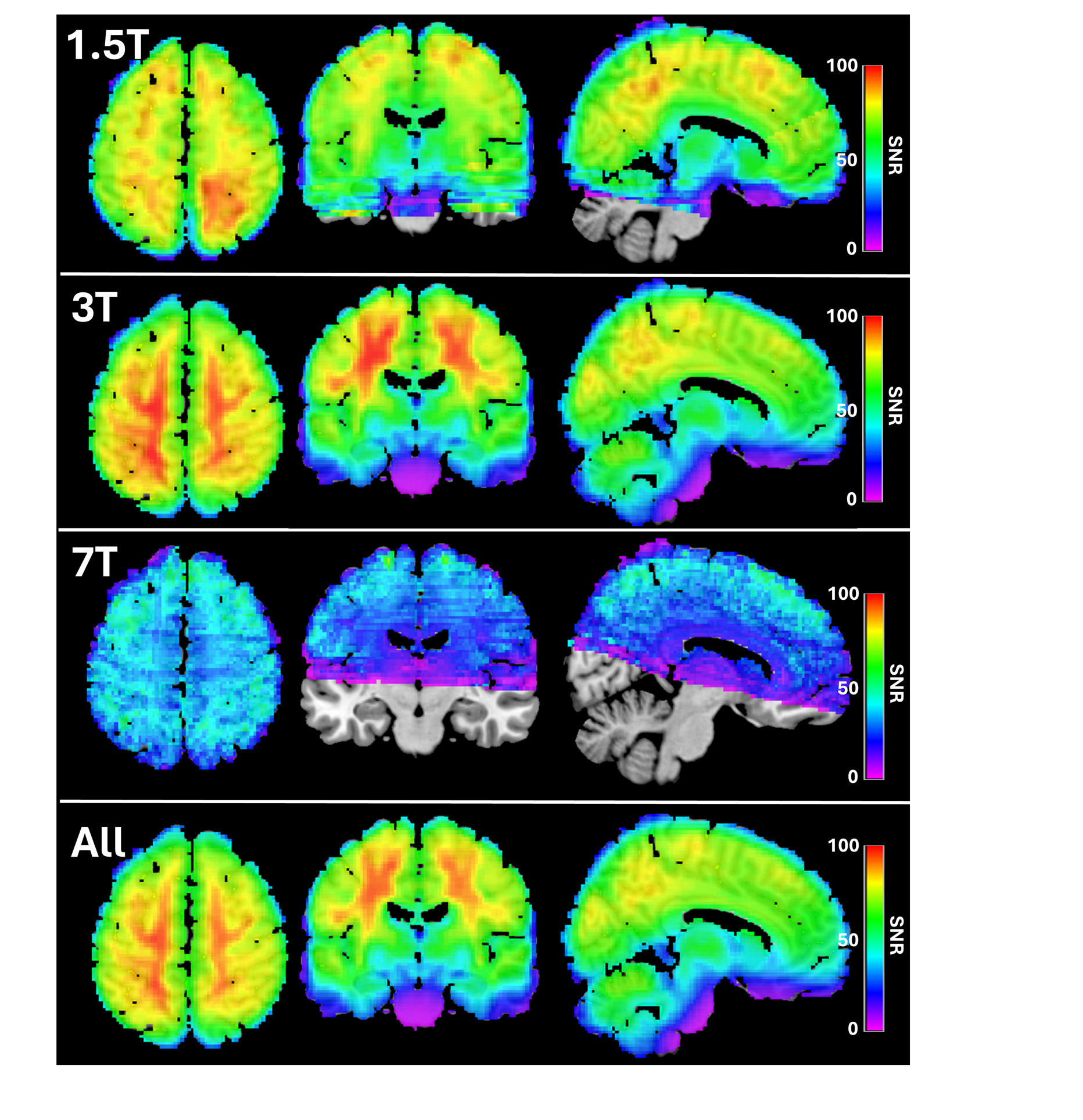
**

**Supplementary Figure S3 – Framewise displacement per task.** Boxplot of the framewise displacement metric per task and per subject averaged over scans with maximum and minimum voxel sizes for the dataset marked by green and red horizontal lines, respectively.


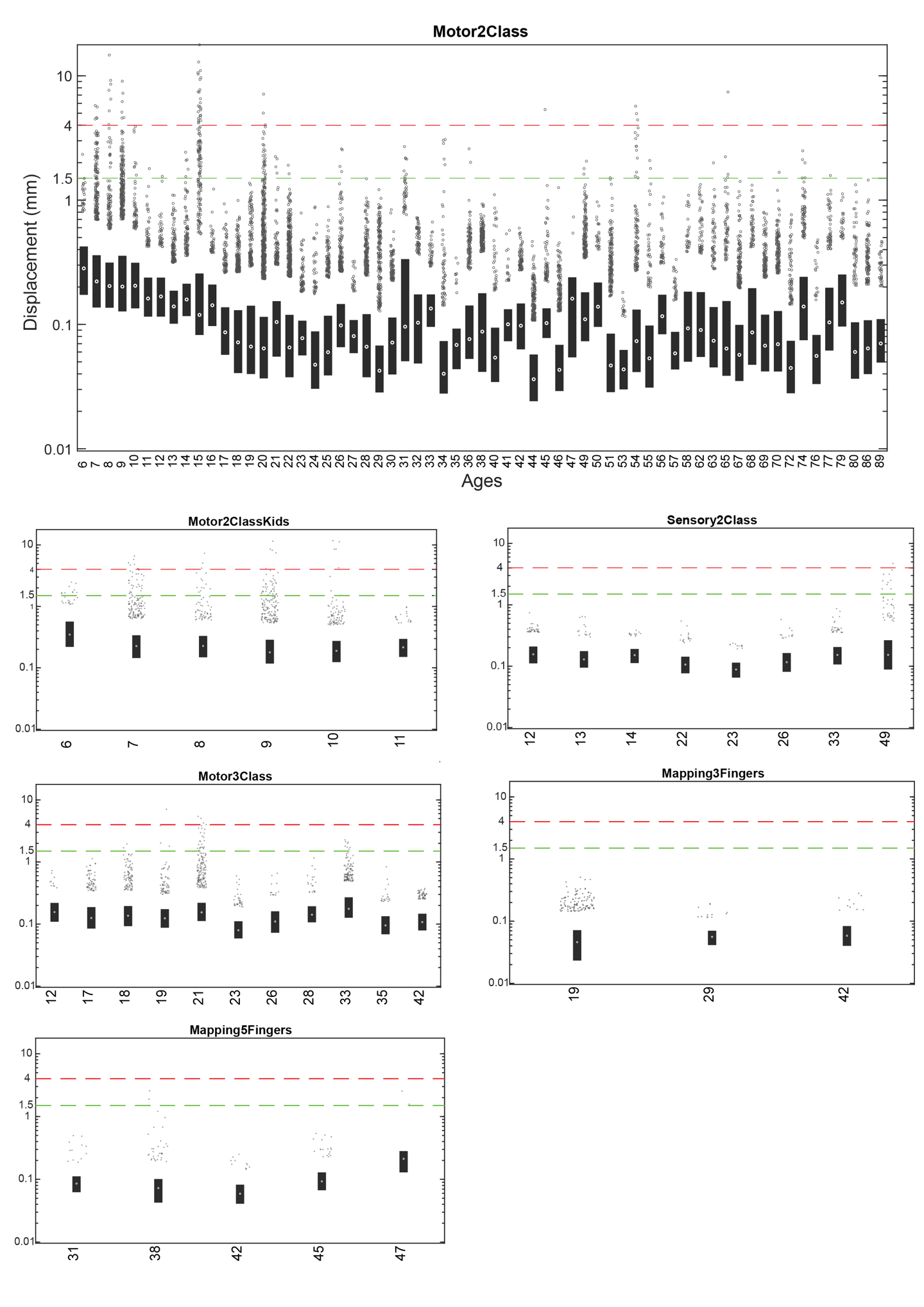

Supplement: Supplementary file 2 — Supplementary Materials [file 41597_2025_5134_MOESM2_ESM.docx]
